# Neuroimaging clues of migraine aura

**DOI:** 10.1186/s10194-019-0983-2

**Published:** 2019-04-03

**Authors:** Nouchine Hadjikhani, Maurice Vincent

**Affiliations:** 1 A.A. Martinos Center for Biomedical Imaging, Massachusetts General Hospital, Harvard Medical School, Boston, USA; 2 Gillberg Neuropsychiatry Center, Sahlgrenska Academy, Gothenburg University, Gothenburg, Sweden; 3 Neuroscience Research, Eli Lilly and Company, Indianapolis, USA

**Keywords:** (3–10): Migraine, Aura, CSD, Brain imaging

## Abstract

While migraine headaches can be provoked, or predicted by the presence of an aura or premonitory symptoms, the prediction or elicitation of the aura itself is more problematic. Therefore, imaging studies directly examining the aura phenomenon are sparse. There are however interictal imaging studies that can shed light on the pathophysiology of the migraine with aura (MWA) cascade. Here, we review findings pointing to the involvement of cortical spreading depression (CSD) and neuroinflammation in MWA. Whether asymptomatic CSD also happens in some migraine without aura is still under debate. In addition, new evidence points to glial activation in MWA, indicating the involvement of astrocytes in the neuroinflammatory cascade that follows CSD, as well as dural macrophages, supporting the involvement of the trigeminovascular system in migraine pain.

## Background

### The symptom spectrum of the migraine aura

Migraine, a partially genetic prevalent brain disorder [1], presents with attacks separated by interictal phases. An attack can be considered as a dysfunctional brain state probably related with long-lasting anatomical and functional consequences, as shown by our group and others [2, 3].

In at least a third of migraine attacks, the headache phase is preceded and/or accompanied by transient neurological symptoms referred to as *aura*. In this case, the condition is classified as migraine with aura (MWA), contrary to migraine without aura (MWoA), when no aura symptom is present [4]. The most frequent aura phenotype consists of positive and/or negative visual phenomena, present in up to 99% of the patients [5]. Phenotypes include primary visual disturbances, for example flashes of light, moving zigzags expanding in a horseshoe pattern, white spots, “like looking through air close to a road paved with asphalt in a very hot sunny day”, colored spots; or less frequent more complex perceptions, such as fractured vision, dysmorphopsias, lilliputian (too small) or brobdingnagian (too large) hallucinations, misperceptions of distances, hemianopsias, tunnel vision, among others [6]. Since visual auras varies in form, severity, and duration, the anatomical location, extent, and probably nature of the underlying occipital dysfunction must vary accordingly.

Auras can present also with sensory (mostly paraesthesias in an upper limb and/or hemiface) or language (mainly paraphasia and anomic aphasia) disturbances, either isolated or (more frequently) in combination with visual phenomena, meaning that other brain structures than those related to visual processing can be affected. When aura involves motor weakness, patients are classified as either sporadic or familial hemiplegic migraine [4]. The underlying dysfunction can affect different areas of the cortex consecutively, as successive aura symptoms can build-up over the course of several minutes. In conclusion, there is a striking inter- and intra-patient variability regarding the presence, nature, pattern, and timing of aura symptoms in migraine [6].

The ICHD-3 defines aura as fully-reversible visual, sensory or other central nervous system symptoms that usually develop gradually in the context of a migraine attack. Three out of 6 features are required: 1) at least one aura symptom spreads gradually over ≥5 min; 2) two or more aura symptoms occur in succession; 3) each individual aura symptom lasts 5–60 min; 4) at least one aura symptom is unilateral; 5) at least one aura symptom is positive; and 6) the aura is accompanied, or followed within 60 min, by headache. Aura types are visual, sensory, speech and/or language, motor, brainstem, and retinal. Typical aura consists of visual, sensory and/or speech/language symptoms by definition [4].

The underlying transitory dysfunction that causes aura can theoretically affect any region of the brain. Because symptoms originated in some areas may be less clear, it is possible some aura-like phenomena apart from typical symptoms do not fulfill the ICHD-3 aura criteria. While visual cortex excitation can be translated into complex and/or intense visual phenomena, it is unclear how other visual phenomena emerge. Likewise, little is known about how possibly aura-related cortical dysfunction would be translated if occurring in areas where neurological signs could be relatively subtle, such as the prefrontal cortex for example. Paucisymptomatic or “symptom-free aura” (i.e., the occurrence of the pathophysiological mechanisms underlying aura but in the absence of symptoms) happening in visual as well as other parts of the brain would lead to the diagnosis of migraine without aura (MWoA). The existence and frequency of “symptom-free aura” in MWoA and MWA are crucial yet unanswered questions. A precise distinction between aura and migraine-related cortical symptoms from distinct pathophysiology cannot yet be established.

Cortical spreading depression (CSD), a slow, self-propagating wave of neuronal and glial depolarization is the neurophysiological phenomenon most likely involved with the pathophysiology of the migraine aura [7, 8], (for reviews see [9, 10]). It is noteworthy that different classes of migraine preventive medications tested chronically dose-dependently reduced the CSD frequency and increased the CSD elicitation threshold [11].

In a study published in 2007 [12], we sent questionnaires to more than 500 migraine patients asking for the presence of symptoms such as difficulties in recognizing faces, colors, the presence of language or memory abnormalities, and other cognitive changes. Remarkably, while 72.2% of MWA patients reported such symptoms, 48.6% of MWoA patients also described symptoms that could be attributed to cortical dysfunctions, potentially linked to cortical spreading depression (CSD) phenomena happening in higher tier areas. In line with common clinical sense, we concluded that neurological symptoms other than the classic aura may be underestimated in migraineurs. It is possible that at least part of these symptoms are produced by CSD-like phenomena in relatively silent areas such as the frontal lobe, where symptoms remain unapparent until lesions grow to a large degree [13]. In support of this possibility are the very recently reported changes in speech patterns prior to migraine attack in individuals diagnosed with migraine without aura, present in close to half of the patients during a migraine attack [14], showing that other periictal symptoms may be more common than previously thought.

### Aura and the various components of the migraine attack

Migraine has different phases, not necessarily evident in all patients – premonitory, aura, headache and postdromic [15]. Evidence suggests that migraine is of neurogenic origin, and that the related vascular events are consequences of neuronal changes [8, 16–19]. Daily imaging of a MWoA patient for 30 consecutive days showed hypothalamic activation as early as 24 h before the migraine pain, suggesting that the hypothalamus could be a generator of migraine attack [20]. Although migraine attacks depend on a susceptible brain, the relative independence between their different components suggests that, rather than a rigid successive chain of events, the migraine episode can be better explained sometimes as a net dysfunction, possibly activated from different places and sequences. Accordingly, the pain may vary in location, the aura can be present or not and start after the pain, the premonitory symptoms can vary, and so on. The aura-headache coupling is particularly puzzling [21–23]. Aura is neither ubiquitous nor phenotypically uniform, even in a single patient. Knowledge about migraine phenotypes and pathophysiology supports the concept that both patterns are possible and not mutually exclusive: a migraine attack may the result of a successive series of events where one necessarily triggers the next, or it may behave as a network of possible brain dysfunctions disrupted in different combinations or sequences.

### Imaging the migraine aura

Key questions involve aura neuroimaging. Are there changes in the brain that accurately reflect the occurrence of aura symptoms? Is the aura CSD hypothesis supported by neuroimaging? If so, are these CSD-like neuroimaging hallmarks present exclusively when aura symptoms are reported? How aura-associated neuroimaging data couple with imaging hallmarks of other migraine attacks phases? And finally, are there detectable changes in the brain that either predispose or appear as a result of migraine aura?

The first report of transient hyperemia followed by spreading decreased cerebral blood flow was described in 1981 by Olesen et al. [24]. They used intra-arterial Xenon133 to show that the concept of migraine aura as a vasospastic event was erroneous (see also [25]). Subsequent studies by the Danish group found results suggestive of spreading oligemia [26, 27] in MWA. It became natural to suppose that the spreading nature of the circulatory changes, mirroring the 2–3 mm/min pace of CSD and not respecting the supply territories of large arteries, would somehow correspond to the CSD phenomenon [7].

In our seminal study (NH) [8], we reported the case of a patient who complained of having migraine auras every time after he had been playing basketball for a sustained amount of time. We therefore decided to invite him to play at the sports facilities located right next to the imaging center, for as long as he thought would be necessary to trigger a migraine aura after stop playing, at which point we would go to the MRI center and image him, showing periods of 32 s of flickering radial checkerboard alternating with 32 s of a black screen with a fixation cross. The participant had a squeeze ball he would press when the aura would start, and when it would end. We repeated this a couple of times, and we also acquired interictal data in order to ensure that we would get a stable signal using this protocol of on/off visual stimulation for a sustained amount of time. Using these datasets, we were able to demonstrate signal changes that contained at least eight characteristics in common between the observed events and animal CSD studies. The fMRI signal in the occipital cortex was time-locked to the onset of the visual aura, the quality of the aura was consistent with the function of the area where it started (area V3a), and its propagation was congruent with the retinotopy of the visual percept. The initial focal BOLD signal increase possibly reflected vasodilation; it progressed contiguously and slowly over the occipital cortex to affect adjacent voxels. After this initial increase, the BOLD signal diminished, (possibly reflecting vasoconstriction following the initial vasodilation), and was then suppressed transiently. During periods with no visual stimulation other than a fixation cross, but during which the subject was experiencing scintillations, the BOLD signal change followed the retinotopic progression of the visual percept, moving from the center of the visual field towards the periphery. We concluded from this study that the data strongly suggested that a propagating event akin to CSD generates the migraine visual aura in human visual cortex.

Clinically, both MWA and MWoA share the same attack features except for aura. They are defined by the same pain characteristics: unilateral, pulsating, and aggravated by physical activity pain lasting 4-72 h, associated with the same symptoms and signs (e.g. changes in appetite, nausea, vomiting, light and noise sensitivity). In addition, they can both be triggered by the same substances (e.g., Nitric Oxide releasers or some neuropeptides) [28], and respond to the same preventive or therapeutic treatments. Symptomatic aura is obviously not obligatory prior to the migraine pain, otherwise neither MWoA nor MWA patients having aura symptoms starting after the headache phase would be possible. In about 20% of cases the headache starts before aura or simultaneously with it [21].

There is evidence to suggest that cortical dysfunction, possibly involving CSD-like phenomena, is also present in migraine without aura [12, 24, 29–31]. Thus, CSD at the same or other cortical sites could be either asymptomatic, evoke typical aura phenomena, or give rise to symptoms not classically recognized as aura. Studies confirming the presence of CSD-like neuroimaging hallmarks in other less typical migraine-generated transitory cortical dysfunctions are not available, although evidence supports the presence of CSD-like phenomena in migraine without aura. As an example, Woods et al. [29] reported in 1994 spreading cerebral hypoperfusion in a patient suffering from migraine without aura, providing the first evidence that CSD may also underlie MWoA – although one should mention that the patient in the report by Woods actually experienced transient visual symptoms (hazy vision). Admitting that CSD is the substrate of aura and possibly occurs in migraine with atypical, subtle, or no aura symptom, the possibility exists that some MWoA patients have the headache associated – or even induced - by relatively silent CSD.

It is noteworthy that ca. 17% of MWA patients may present prolonged episodes [32]. Neuroimaging evidence linking this aura phenotype to CSD-like changes in humans is lacking. In the KCl stimulated gyrencephalic feline cortex, secondary CSD events were shown to propagate in parallel to the gyrus in which they took place originally, encompassing a significantly smaller cortical area and propagating with a significantly slower velocity [33]. It remains to be demonstrated whether secondary or parallel CSD waves in the human cortex could manifest as prolonged aura in migraineurs. The fact that migraine aura is characterized by symptoms that can start in succession (with or without a symptom-free interval) or simultaneously indicated that either multiple CSD waves arise at different points in topography and time, or waves may travel asymptomatically across cortical areas producing later symptoms at regions distant from the cortex originally symptomatic [6].

CSD is accompanied by matrix metalloproteinase-9 (MMP-9) activation and neurogenic inflammation [34, 35]. Indirect evidence of neuroinflammation has been shown in migraine, with intracranial plasma extravasation (one case report, [36]), and gadolinium enhancement (one case report, [37]). Despite a lot of efforts, it has been difficult to reliably demonstrate gadolinium enhancement in migraine. Some studies have used superparamagnetic iron oxide nanoparticles (ferumoxytol) to assess areas of blood brain barrier (BBB) dysfunction during neuroinflammation [38]. These particles are taken up by many elements of the immune system, including the microglia – however, their size comparable to viruses, makes them less likely to cross the BBB, supposedly intact in MWoA [39] and MWA [40], although increased levels of matrix metalloproteinase-9 (MMP-9) have been associated with migraine [41, 42]. It is possible that the techniques used so far have not been sensitive enough to definitely objectify BBB disruption in migraine.

Animal models of CSD indicate that neuroinflammation and microglia activation may be key factors in the generation of the pain associated with migraine [43, 44]. A study in rodents has demonstrated that a complex cascade induced by CSD leads to neuroinflammation, including astrocytic and microglia activation [45], eventually leading to pain via activation of the trigeminovascular system. That study demonstrated that CSD leads to Pannexin 1 channels opening and activation of pro-inflammatory mediators, that in turn induce cyclooxygenase-2 and inducible Nitric Oxide synthase expression in astrocytes, and microglia activation. Astrocyte release of cytokines, prostanoids and Nitric Oxide in the subarachnoid space promote sustained activation of the trigeminal nerve fibers surrounding pial vessels, and the trigeminal nerve collaterals innervating the middle meningeal artery initiate neurogenic inflammation, including mast cells degranulation.

We recently used combined PET/MR imaging techniques with [11C]PBR28, a radioligand that binds to the 18 kDa translocator protein, a marker of glial activation, in migraineurs with visual aura who had experienced one or more migraine episodes during the two weeks preceding the imaging session [46]. We observed increased signal in areas previously shown to be involved in CSD generation (primary visual cortex, visual areas V3A and MT, and Broca’s area) as well as with areas involved in pain processing (thalamus and primary/secondary somatosensory and insular cortices). Increase of the signal was also positively associated with the frequency of migraine attacks. We also found increased signal in the frontal pole and the orbitofrontal cortex, areas in which CSD may produce symptoms that may be difficult to notice or to interpret. In that same group of patients, we also observed increased uptake of [11C]PBR28 in the meninges, possibly reflecting activation of dural macrophages following MWA (Hadjikhani et al., in preparation).

In migraine, several studies have pointed to neuroinflammation as a possible substrate for the generation of pain [47, 48], after the release of proinflammatory peptides subsequent to CSD [34].

CSD has been shown to induce pial vasodilation involving the release of CGRP [49]. The CSD-related delayed hyperemia is abolished by transecting trigeminal and parasympathetic fibers [34]. Olcegepant and other small molecule CGRP antagonists, as well as CGRP and CGRP receptor antibodies have shown to modulate CSD [50]. On the other hand, the CGRP antagonist BIBN4096 did not block in rats the CSD-induced activation of meningeal afferents, suggesting that a CSD-evoked headache involves other mediators [51].

CGRP, a vasodilating neuropeptide peripherally released by trigeminal fibres, was found increased in the jugular blood ipsilaterally to the head pain during a migraine attack [52], and is reduced in parallel with headache lowering following sumatriptan treatment [53]. New effective anti-migraine treatments block CGRP signaling [54].

In the animal model, genetically driven cortical hyperexcitability predispose to CSD [55]. Hyperexcitability of the central nervous system has been speculated in both MWA and MWoA [56–58], and our group has reported microstructural changes in the thalamus, an important structure in the control of cortical excitability, in migraineurs [59].

Repetitive episodes of neuronal inflammation may result in retrograde degeneration of the neurovascular unit, with as a consequence small lesions in the brain [60]. Migraine has indeed been linked to silent infarct-like lesions (identified by magnetic resonance imaging (MRI) regardless of clinical manifestations) [61–63] that may be triggered by vascular changes linked with inflammation. However, in a follow-up of the original CAMERA study, MWA was not associated with white matter lesions [64], and a large population-based study with female twins did not confirm an increased risk of silent infarcts in migraine with aura [65]. Other structures seem to be affected by **r**epetitive migraine attacks including the thalamus [59], the frontal lobe and the cerebellum [66]; and cortical thickness increases in the somatosensory cortex and in extrastriate visual areas have been reported [67–69]. Notably, Gaist et al. [70]. recently reported increased cortical thickness in a large group of MWA patients compared to controls, confirming our previous findings [68], but Hougaard et al. [71] found no increase of somatosensory cortical thickness in MWA patients with somatosensory aura, suggesting that structural changes (increased neuronal density?) in visual cortical areas predispose to CSD, but that CSD per se does not induce increased cortical thickness. In addition, our recent studies have also shown alterations of brain function, with increased connectivity in the pain matrix [72, 73]. It is also known that repetitive episodes of pain also lead to allodynia, reflecting hypersensitivity of the somatosensory system [74–76]. Whether long-term maladaptive plastic changes are at the basis of the chronification in migraine, which happens at a yearly rate of about 3% needs to be further explored [77].

Other conditions such as occipital epilepsy can mimic migraine aura [78–80], although seizures tend to be shorter, occur in clusters, sometimes developing into temporal lobe or generalized epilepsy; however the differential diagnosis may be complicated by the fact that they can be accompanied by migrainous headache. Human neuroimaging depicting CSD-like phenomena in connection to seizures is lacking. The actual role of CSD in epilepsy is complex and obscure as data suggest that CSD can predispose to epileptic activity and vice-versa [81]. Migraine aura can be mistaken for a stroke [82, 83] as areas of hypoperfusion can be evidenced during migraine aura. Transient global amnesia (TGA), manifested as a transitory memory loss, could be a manifestation of hippocampal CSD, but may as well be due to a transient ischemic attack or a stroke, and abnormalities in diffusion weighted imaging can be observed due to either etiologies [84], rendering the differential diagnosis even more difficult.

Although not yet reproduced by similar studies, hypoperfusion of the cerebellum and crossed cerebellar diaschisis, an associated hypoperfusion of the frontal cortex in the opposite hemisphere, were reported in patients with MWA. Because it is not accompanied by severe diffusion imaging abnormalities nor cerebellar infarction, even in those who have prolonged symptoms for up to 24 h, it may be considered a benign phenomenon [85].

## Conclusion

In conclusion, neuroimaging data indicate that: 1) migraine aura is related to a CSD-like phenoma in MWA patients; 2) spreading phenomena similar to CSD may occur in MWoA, suggesting that either CSD can be asymptomatic, that symptomatic aura depends on other factors than just CSD, or that CSD in MWA differs fundamentally from the spreading oligoemia found in MWoA; 3) visual aura most probably starts at visual cortical areas such as V3A and MT; 4) glial activation is present following migraine attacks at areas previously shown to be involved with aura generation and/or pain processing, increasing in accordance to the headache frequency; 5) activation of meningeal macrophages in MWA further support the activation of the trigeminovascular system by CSD. It remains to be explained how the aura phenotype varies vastly even when the same cortical areas are involved; to which extent CSD happens in MWoA and what makes it phenotypically apparent, and how a CSD-like phenomenon as demonstrated by neuroimaging couples mechanistically with the other phases of the migraine attack.

## Abbreviations

BBB: Blood brain barrier
BOLD signal: Blood-Oxygen-Level Dependent signal
CSD: Cortical spreading depression
fMRI: Functional Magnetic Resonance Imaging
MMP-9: Metalloproteinase-9
MWA: Migraine with aura
MWoA: Migraine without aura
PET: Positron Emission Tomography
